# Antibiotic Resistance Genes Carried by Commensal *Escherichia coli* from Shelter Cats in Italy

**DOI:** 10.3390/vetsci10120680

**Published:** 2023-11-28

**Authors:** Delia Gambino, Francesco Giuseppe Galluzzo, Luca Cicero, Roberta Cirincione, Erika Mannino, Veronica Fiore, Daniela Proverbio, Eva Spada, Giovanni Cassata, Valeria Gargano

**Affiliations:** 1 Istituto Zooprofilattico Sperimentale della Sicilia “A. Mirri”, 90129 Palermo, Italy; deliagamb@gmail.com (D.G.); robertacirincione@gmail.com (R.C.); manninoerika@gmail.com (E.M.); veronicafiore@hotmail.it (V.F.); giovanni.cassata@izssicilia.it (G.C.); valeria.gargano@izssicilia.it (V.G.); 2 Department of Veterinary Medicine and Animal Sciences (DIVAS), University of Milan, 26900 Lodi, Italy; daniela.proverbio@unimi.it (D.P.); eva.spada@unimi.it (E.S.)

**Keywords:** *Escherichia coli*, antimicrobial resistance, stray cats

## Abstract

**Simple Summary:**

The epidemic of antimicrobial resistance is a widespread health challenge that deserves a One Health approach. Bacteria resistant to antimicrobials and their resistance genes can be transferred from food-producing animals and pets to humans and vice versa. Many studies have shown that resistant bacteria are emerging in companion animals and that a number of resistance genes are being shared between pets and humans. Even stray cats, which have contact with humans and share the urban environment with them, can therefore act as reservoirs of antimicrobial resistance for humans and their pets. Therefore, to investigate the implication of these animals as disseminators of antibiotic resistance, we phenotypically and genotypically assessed the resistance of commensal *E. coli* isolated from stray cat feces. The *E. coli* analyzed were resistant to ampicillin, tetracyclines and sulfisoxazole and carried genes that encode these resistances. Even though there is still a need for further studies, the occurrence of resistant *E. coli* provides support for the assumption that stray cats may be fecal sources of resistance, so it is necessary to monitor these animals in antimicrobial resistance surveillance programs.

**Abstract:**

Antimicrobial resistance is a widespread global health problem. The presence of resistant bacteria and antibiotic resistance genes has been demonstrated not only in humans but also in animals, including pets. Stray cats share the urban environment with people and pets. This may facilitate transmission of resistant bacteria and resistance genes between stray animals, people and domestic animals. Several studies have investigated the role of stray cats as a fecal carrier of ESBL-producing bacteria. However, there are many genes and resistance mechanisms that can be detected in commensal *E. coli*, which, because of its genetic plasticity, is considered an indicator for monitoring antibiotic resistance. In this study, rectal swabs were collected from stray cats from colonies and shelters in the city of Monza (Monza Brianza, Italy) to isolate commensal *E. coli*. Phenotypic tests, such as the minimum inhibitory concentration (MIC) and the double disc test (DDST), and molecular analyses to detect antimicrobial resistance genes (ARGs) were used to study the resistance of these isolates. The results obtained confirm that stray cats can carry ESBL-producing *E. coli* (6.7%) and genes conferring resistance to other important antibiotic classes such as tetracyclines and sulfonamides.

## 1. Introduction

*Escherichia coli* is a widely disseminated bacterium of the *Enterobacteriaceae* family. While pathogenic *E. coli* strains can cause infection in humans and animals, most strains colonize the intestine in a harmless way and only occasionally cause disease in healthy individuals [1]. However, non-pathogenic *E. coli* isolates show an increased ability to acquire virulence factors and resistance to various antimicrobials, as well as an efficient transmission and colonization capacity. The *E. coli* genome is characterized by the presence of mobile genetic elements such as insertion sequences (IS), integrons, plasmids and transposons, which promote horizontal gene transfer (HGT) between this species and other bacteria. Virulence genes are mostly associated with IS, whereas antimicrobial resistance genes (ARG) are mostly carried on plasmids, transposons and integrons (class 1, 2 and 3 integrons), and all these elements play a central role in rearrangement and gene transfer. Integrons, in particular, are able to extract ARGs from their environment and assemble them in their gene cassettes through specific recombination. This means that in the intestine, where bacterial population density and species diversity are high, commensal *E. coli* can acquire ARGs and transfer them to other commensal strains, as well as pathogens acting as an ARG reservoir [2]. Resistant *E. coli* strains are widely distributed, and the last decade has seen an increase in resistance, especially to certain classes of antibiotics such as beta-lactams and fluoroquinolones [3].

In Europe, the surveillance of antimicrobial resistance in commensal indicator *E. coli* isolated from the intestinal microbiota of healthy livestock serves as a valuable means to track the dissemination of resistant bacteria. Notably, both antimicrobial resistance bacteria (ARBs) and ARGs have the potential to be transferred between animal and human populations, thereby posing a risk for the transmission of resistance genes to pathogenic bacteria in both humans and animals [4]. The high levels of antimicrobial resistance detected among isolates from food-producing animals have highlighted the need for cross-sector collaboration between the human, veterinary and food production sectors as part of a “One-Health” strategy [5]. Although many previous publications have revealed the prevalence among pets of multi-resistant pathogens similar to those identified in humans, such as Gram-negative extended spectrum beta-lactamase producers (ESBL-producers), AmpC-type beta-lactamases or carbapenemases [6,7,8,9], demonstrating the existence of an emerging health problem, these animals are not included in systemic antimicrobial resistance monitoring programs [10]. Moreover, most data on antimicrobial resistance in pets refer to household pets, and focus on pathogenic bacteria and specific sites of infection (urinary tract, skin, ear, and gastrointestinal infections) [1,11,12,13]. Close contact between pets and owners, who share a home environment and have contact with the same surfaces and objects, can promote interspecies transmission of resistant bacteria [14,15,16]. Stray cats are synanthropic animals that meet and interact with humans in the urban environment and may also act as reservoirs of AMR. The urban environment is an extremely complex network in which factors, such as high human density, the presence of other animals and numerous microenvironments (buildings, open spaces, parks, sewers), provide opportunities for selection and transport of ARBs and ARGs [17]. Strays living in these environments can therefore acquire ARBs and ARGs from various urban sources (soil, garbage, feces of other animals, and sewers) and transmit them to humans, either indirectly, by sharing the urban environment, or directly, in the case of volunteers caring for them in shelters or colonies or owners adopting them.

In Italy, the count of stray cats is conducted based on the number of sterilizations performed by the national health system within trap, neuter and release (TNR) sterilization programs. These data are provided by each Italian region and published annually by the Ministry of Health, whose latest report for 2021 showed 11,228 stray cats sterilized in Lombardy. The Stray Animal Law requires that stray cats be trapped and transferred to shelters where they are sterilized and have all the necessary care before they are either adopted (if suitable) or returned to their colonies. Cat colonies are groups of cats living free in urban areas, usually at the same locations, and managed by animal welfare organizations or private volunteers [18]. In this context, the possibility of these animals coming into contact with humans, such as volunteers (who care for them in shelters or colonies) or new owners (in case of adoption), and transmitting ARBs or ARGs to them, should not be overlooked.

The aim of this work was to evaluate the role of stray cats as reservoirs and fecal vectors of ARBs or ARGs. In order to do this, phenotypic and molecular tests were carried out on commensal *E. coli* isolated from stray cats housed in animal shelters or belonging to cat colonies in the city of Monza (Monza Brianza, Italy).

## 2. Materials and Methods

### 2.1. Escherichia coli Strains Collection

A total of 60 rectal swabs from stray cats were analyzed in order to isolate *E. coli* strains and assess their phenotypic and genotypic antibiotic resistance. Sampling was performed in 2022 during activities related to the SARS-CoV-2 infection surveillance in stray cats in the city of Monza, Monza Brianza, in the Lombardy region, northern Italy.

Rectal swabs were collected from n = 35 cats from nine different colonies in the province of Monza Brianza (n = 7 from a colony of Monza, n = 6 from Brugherio, n = 5 from Muggiò, n = 4 from Agrate Brianza, n = 4 from Cornate D’Adda, n = 4 from Lissone, n = 3 from Cernusco sul Naviglio, n = 1 from Concorezzo and n = 1 from Caponago) and n = 25 from the ENPA shelter in Monza Brianza (Lombardy, Italy), which were captured and received general anesthesia to perform neutering surgery as part of trap, neuter and release (TNR) sterilization programs. TNR programs are carried out as part of a national program to control stray pet populations under Italian National Law (law no. 281/1991). Samples were collected with the informed consent of those legally responsible for the stray colonies or shelter cats, and in accordance with the study and animal welfare protocol, which was revised and authorized by the Animal Welfare Bioethical Committee of the University of Milan (approval number OPBA _91_2020, released on 15 January 2021 and OPBA_34_2021, released on 12 March 2021).

After collection, rectal swabs were stored at -4 °C and sent within 24–48 h to the Istituto Zooprofilattico Sperimentale (IZS) of Sicily (Palermo, Italy), where they were seeded on McConkey agar plates (Oxoid, Milan, Italy) incubated at 37 °C for 24 h. One or two colonies with morphology attributable to *E. coli* were selected from each plate, if available, and purified on brain heart infusion agar (Oxoid, Milan, Italy) incubated at 37 °C for 24 h. The isolated and purified strains were subjected to biochemical–enzymatic screening tests (citrate production, urea, H_2_S, glucose and lactose fermentation) to discriminate *E. coli* from other enterobacteria. DNA of each strain was extracted with an automated King Fisher extractor (Thermo Fisher Scientific, Waltham, MA, USA) using the QIAamp One-For-All Nucleic Acid Kit as recommended by the manufacturer (QIAGEN Sciences, Germantown, Maryland, USA). Before performing the PCRs, DNA quality and concentrations were assessed using NanoDrop™ 8000 Microvolume UV-Vis spectrophotometer (Thermo Fisher Scientific, Inc., Wilmington, DE, USA).

PCR was carried out using a Platinum™ Taq DNA Polymerase High Fidelity (Thermo Fisher Scientific, MA USA) and following the factory instruction. In order to identify the strains, 16S ribosomal gene amplifications were then performed, as reported by Li et al. [19]. Briefly, the reaction mix was prepared with 0.4 mM of forward and reverse primer, 1X high fidelity PCR buffer, 2 mM MgSO_4_, 0.2 mM of each dNTP, 0.2 of forward and reverse primer, 10 ng of genomic DNA and autoclaved distilled water to 50 μL. The amplification reaction involved an initial denaturation at 94 °C for 1 min and then 35 cycles consisting of denaturation for 1 min at 94° C, annealing for 1 min at 55 °C and extension for 1 min at 68 °C. In each PCR run, *E. coli* ATCC 25,922 (American Type Culture Collection, Rockville, MD, USA) was used as a positive control; nuclease-free water was used as the negative one. Subsequently, 5 µL of each amplification reaction was analyzed by electrophoresis analysis using E-Gel™ Go! Agarose Gels, 2% (Thermo Fisher Scientific, Waltham, MA, USA) to determine product size. Finally, the PCR products were purified and sequenced at BMR Genomics Srl (Padua, Italy).

### 2.2. Determination of Minimum Inhibitory Concentration (MIC)

The antibiotic susceptibility of *E. coli* isolates was assessed using the minimum inhibitory concentration (MIC) method. Using commercial plates (Thermo Scientific 96-well Sensitititre™ Plate, Waltham, MA, USA), the MIC values (μg/mL) of 10 antibiotics were determined. The antibiotics and their dilutions tested were amoxicillin/clavulanic acid (0.25/0.12–32/16 μg/mL), ampicillin (0.25–32 μg/mL), cefazolin (0.5–8 μg/mL), cefotaxime (0.5–4 μg/mL), colistin (0.03–8 μg/mL), enrofloxacin (0.03–32 μg/mL), gentamicin (0.25–32 μg/mL), sulfamethoxazole/trimethoprim (0.06/1.19–16/304 μg/mL), sulfisoxazole (128–512 μg/mL), and tetracycline (0.5–16 μg/mL). After preparing a bacterial suspension with 0.5 McFarland turbidity in 5 mL of sterile water, 10 μL of this was mixed in 10 mL of Mueller–Hinton broth (Thermo Fisher Scientific, Waltham, MA, USA). Subsequently, 50 μL of inoculated broth was dispensed into each well of the MIC plate, which was incubated at 37 °C for 18–24 h. Manual plate reading was conducted with the Sensititre™ Manual Viewbox (Thermo Fisher Scientific, Waltham, MA, USA) and results were interpreted according to Clinical and Laboratory Standards Institute (CLSI) breakpoints [20].

### 2.3. Double Disk Sinergy Test

The double-disk synergy test (DDST) was performed as recommended by EUCAST [21]. Briefly, a 0.5 McFarland bacterial suspension was seeded on Muller-–Hinton agar (Oxoid, Milan, Italy) and three discs containing cephalosporins (cefotaxime 30 µg, ceftazidime 30 µg, cefepime 30 µg) were positioned beside a disc containing clavulanic acid (amoxicillin–clavulanic acid 30 µg). The test was interpreted as positive if there were increased zones of inhibition around the cephalosporin discs or a “keyhole” towards the amoxicillin–clavulanic acid disc.

### 2.4. Multiplex Real-Time PCR for ESBLs Determination

A multiplex Real-Time PCR was performed to assess the presence of resistance genes to extended spectrum beta-lactamase antibiotics. Therefore, DNA obtained from each strain was subjected to a Real-Time PCR analysis specifically for the determination of the ESBL phenotype [22]. Real-time amplifications were performed in 25-µL reactions containing 12.5 µL of SsoAdvanced Universal Probes Supermix (Bio-Rad Laboratories S.r.l., California, USA), 0.4 µM of each forward and reverse primer (Table S1), 0.2 µM of TaqMan *blaTEM* probe, 0.4 µM of each of the other four TaqMan probes, 0.6 µL of sterile water, and 10 pg of DNA. Positive controls consisted of four ATCC^®^ (Manassas, Virginia, USA) strains (*E. coli* BAA-3048™, *E. coli* BAA-3049™, *E. coli* BAA-3051™ and *Klebsiella pneumoniae* BAA-3060™, American Type Culture Collection, Rockville, MD, USA) harboring the searched genes, while DNAase- and RNAase-free water was used as a negative control.

### 2.5. Class 1 Integron and ARGs Detection for Tetracyclines, Sulfonamides and Fluoroquinolones

A reaction mix containing a concentration 1X of 5X Platinum II PCR Buffer, 10 mM dNTPs, 0.5 μM of each primer of the pairs shown in Table S2, 1.25 U of Platinum II Taq DNA Polymerase DNA polymerase (Thermo Fisher Scientific, MA USA), 10 ng of genomic DNA and nucleus-free water to obtain a volume of 50 μL was prepared. For each PCR reaction, DNA from two ATCC^®^ (*E. coli* BAA-3048™ and *E. coli* BAA-3051™) strains harboring the researched genes was used as positive controls and DNAase- and RNAase-free water as a negative control. The size of all amplicons was verified by electrophoresis on E-Gel™ Go! agarose gel, 2% (Thermo Fisher Scientific, Waltham, MA, USA).

### 2.6. Statistical Analysis

Statistical analyses were conducted with R version 4.3.1 (16 June 2023). The phenotypic resistance variables were correlated with the genetic resistance to the antibiotics (resistance to beta-lactam, tetracyclines, sulfonamides, and fluoroquinolones). Fisher’s exact probability test was used to evaluate if there is an association between phenotypic and genetic resistance. The p-value was considered as significant if *p* <0.05.

## 3. Results

### 3.1. Phenotypic Profile

Phenotypic tests were carried out on the 60 *E. coli* isolates to determine the susceptibility to 10 antibiotics and the occurrence of ESBL-producing isolates. Table 1 shows the MIC values obtained for the 60 *E. coli* analyzed in this study.

Among the *E. coli* isolates, 40% (24/60) were resistant to at least one of the investigated antibiotics. Four isolates were multi-drug resistant (MDR), as one isolate showed resistance to four classes of antibiotics (beta-lactams, colistin, tetracyclines and sulfonamides) and three other isolates to three classes, i.e., beta-lactams, tetracyclines and sulfonamides. No strains showed resistance to enrofloxacin and gentamicin, but resistance was found to the other eight antibiotics tested (Table 1). The most common resistances were to ampicillin (20%), sulfisoxazole (18.3%), tetracycline (18.3%) and cefazolin (13.3%). Instead, less than 10% of the strains showed resistance to amoxicillin/clavulanic acid, sulfamethoxazole/trimethoprim, cefotaxime and colistin.

The DDST test was performed on all strains and allowed to identify 4/60 strains with an ESBL profile, the same strains that showed resistance to cefotaxime.

### 3.2. Genotypic Profile

The ARGs detection conducted on the 60 *E. coli* isolates showed that 31.6% (19/60) harbored at least one ARG among those screened for. The gene most frequently detected was *tet*(*A*), present in 16.7% (10/60) of isolates, followed by *sul1* (13.3%, 8/60) and *tet*(*B*) (10%, 6/60). Also, although with low frequency, *bla_TEM_* (8.3%, 5/60) and *bla_CTX-M_* (6.7%, 4/60) genes were also detected. No strains were found to harbor the *bla_SHV_*_,_ *bla_CMY_, sul2*, *gyrA* and *parC* genes. Table 2 shows the phenotypic and corresponding genetic profiles detected in the tested isolates.

### 3.3. Data Analysis

**Figure 1:**
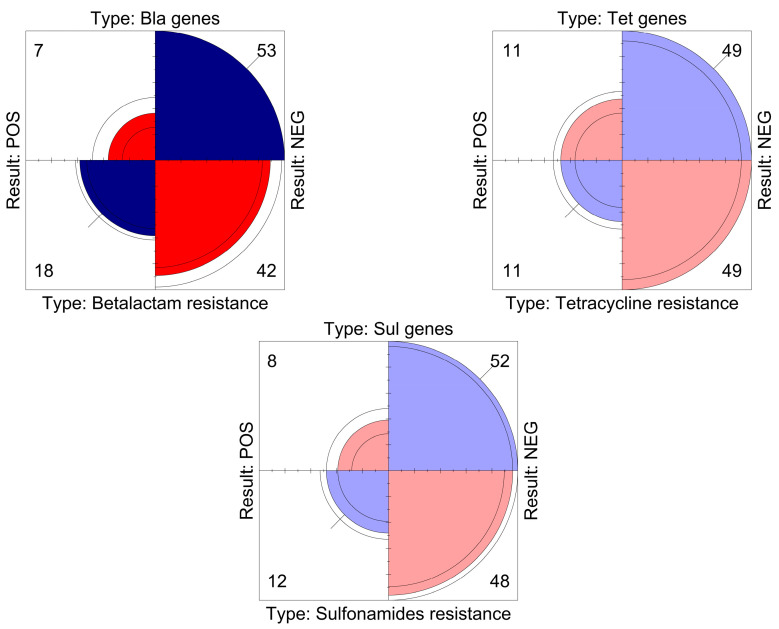
The graph displays, for each antibiotic class, a quadrant view of the relationship between the phenotypic resistance exhibited by the strains and the genes that were investigated. The area of each quadrant is proportional to the frequency of positives/negatives detected, indicated numerically in each corner.

Statistical analysis showed that beta-lactams, tetracyclines and sulfonamides had a significant correlation between the phenotypic resistance detected and the genes investigated (*p*-value < 0.05) (Figure 1). This correlation was also significant for fluoroquinolones, for which no discrepancies were found.

## 4. Discussion

The transmission of antimicrobial-resistant bacteria or their resistance genes to humans can occur from both animals for food production and pets. Many studies have shown the attendance of resistant bacteria in pets and that several ARGs are spread between these animals and humans [6,7,23]. Stray cats can also act as reservoirs of AMR, as they can transmit ARBs and ARGs to humans and their pets but can also acquire them [6,7,8,9]. These animals live in urban environments where the conditions are favorable for the selection of resistance. Urban environments are characterized by the presence of selective pressures (antibiotics, heavy metals and biocides), high bacterial diversity and human density [17]. These factors, combined with the characteristics of bacteria, such as their ability to respond to stress, to implement mechanisms to minimize the fitness cost of ARGs, to form biofilms and, above all, the genetic plasticity of bacteria, are all factors that help favor the selection of resistant bacteria and resistance genes. As a consequence, stray cats living in this environment can acquire selected ARBs and ARGs and become reservoirs and disseminators of resistance. They can spread ARBs and ARGs in the environment through feces and, in countries such as Italy where stray animal management and adoption is encouraged, they can also spread them through direct contact with volunteers in shelters and colonies or, in the case of adoption, with owners.

In this study, we investigated the resistance of commensal *E. coli* isolated from rectal swabs of stray cats to assess the potential role of these animals as reservoirs and disseminators of AMR. Phenotypic and molecular tests were performed on the 60 isolates to determine their resistance, ESBL production and the presence of selected ARGs. The MICs showed that these isolates were resistant to three classes of antibiotic: beta-lactams (30%), sulfonamides (20%) and tetracyclines (18.3%). Looking at individual molecules, our results are consistent with those reported in other studies conducted on domestic and stray cats in Italy [15,23,24]. The highest resistance rates were found for ampicillin, sulfisoxazole and tetracyclines. These results are consistent with those reported in previous studies on stray and domestic cats [6,7,24]. Regarding sulfonamides, a higher resistance was found for sulfisoxazole (18.3%) and a lower for trimethoprim/sulfamethoxazole (6.7%), the latter also being lower than in the above-mentioned studies where the percentage of resistant strains was at least 33.6% [7,24]. The ESBL phenotype was detected in 6.7% of isolates by the DDST test, confirming that stray cats can harbor ESBL-producing bacteria even at low prevalence, which is in agreement with previous studies [25]. This result was confirmed by molecular analysis, which detected the presence of the *bla_CTX-M_* gene in our four ESBL-producing isolates. Although *bla_CTX-M_* gene variants were not tested in this study, it is known that all *bla_CTX-M_* variants encode extended spectrum beta-lactamases that confer resistance to most beta-lactam antibiotics, including third-generation cephalosporins. These genes are widely distributed internationally and have been detected in clinical ESBL isolates from humans and in isolates from several animal species, including dogs and cats [25,26]. Among the *bla* genes, the presence of *bla_TEM_* (8.3%) has also been detected. There are now over 200 known variants of this gene, with different levels of resistance (narrow and extended spectrum) [27]. Although the variants were not determined in this study, the occurrence of the *bla_TEM_* gene in isolates from dogs and cats, including strays, has been investigated and reported by other authors, confirming the possibility that these animals are not only carriers of resistant bacteria but also of the *bla* gene with its different spectrum of activity [7,9,25,28].

While nineteen *E. coli* isolates harbored one or more genes that could be involved in the phenotypic resistance they exhibited, this was not the case for five isolates in which no ARG was detected. Resistance to antimicrobials can be mediated by several major mechanisms encoded by numerous genes and variants. Therefore, it is possible that in these five *E. coli* isolates, the genes responsible for resistance are different from those we investigated [29]. In this study, the most frequently detected genes were the *tet* and *sul* genes. With regard to tetracycline resistance genes, the *tet*(*A*) gene was the predominant resistance determinant (16.6%), followed by the *tet*(*B*) gene (10%). Notably, the *tet*(*A*) gene was found in all tetracycline-resistant strains. The detection of the *tet*(*A*) gene suggests that active efflux is the main mechanism of tetracycline resistance in these *E. coli* isolates from stray cats, which is consistent with the epidemiological trend of tetracycline resistance genes in *E. coli* of animal origin [30,31]. The *sul1* gene was detected in 13.3% of *E. coli* isolates that also harbored the class 1 integrons. Sulfonamides resistance is widespread in Gram-negative bacteria from animals and humans worldwide [32]. The *sul1* is one of the genes coding for resistance to sulfonamides and was found almost exclusively on large conjugative plasmids and class 1 integrons. This class of integrons is the most abundant and clinically relevant and plays a crucial role in the spread of AMR genes [33].

The presence of ARGs in association with class 1 integrons in *E. coli* isolates from stray cats suggests that the discovered genes are organized in gene cassettes and could be transferred to other bacteria via HGT, which would contribute to the spread of resistance. Indeed, gene cassettes can be transferred from one bacterium to another via integrons. The acquisition, transfer and maintenance of class 1 integrons is thought to be one of the causes of the steady increase in the emergence of resistant *E. coli* over time [34].

## 5. Conclusions

In Europe, antimicrobial resistance surveillance programs only consider food-producing animals, and although the European Medicines Agency (EMA) monitors the commercial sales and consumption of antimicrobial products in animals, data on companion animals are not included in the annual reports of either the EU or the World Organization for Animal Health (WOAH). Antimicrobial resistance is now recognized as a global health issue that deserves a “One Health” approach, but the AMR surveillance programs implemented by many countries do not include domestic and stray animals, which can be a source of ARBs and ARGs. Our results, which are consistent with previous studies, confirm the carriage of not only ESBL-producing *E. coli* but also ARGs for tetracyclines and sulfonamides in the feces of stray cats, highlighting their potential role as reservoirs. Although this study has its limitations due to the lack of information on stray cats (age, permanence in shelter, clinical history, antimicrobial treatments, environmental contamination status, direct contact with humans and animals), the results obtained highlight the importance of including stray cats in surveillance programs, both to assess the prevalence of antimicrobial resistance and as possible sources for the spread of antimicrobial resistance. Stray cats are reservoirs and sentinels for the spread of AMR in city environments and therefore deserve to be controlled, but monitoring these animals is certainly not easy. Useful data on stray cats could be collected as part of TNR activities carried out in many European countries, including Italy, and would be useful for the implementation of AMR surveillance systems. Furthermore, since stray cats can be carriers of ARBs and ARGs, it would be appropriate to train and educate volunteers who care for them. For this purpose, the recommendations issued by the Italian Ministry of Health together with the National Plan to Combat Antimicrobial Resistance 2020–2025 are useful. In addition to emphasizing the need to prescribe and use antimicrobials correctly, these recommendations underline the need for appropriate hygiene and preventive measures to control infections and the spread of ARBs.

## Figures and Tables

**Table 1:** MIC values detected.

Antimicrobial Agent	Number of Isolates at the Indicated MIC Value (µg/mL)															S	R
	0.03	0.06	0.12	0.25	0.5	1	2	4	8	16	32	64	128	256	512	(%)	(%)
Amoxicillin/clavulanic acid				1	1	15	10	17		1	**5**					91.7	8.3
Ampicillin				3		12	16	15	2		**12**					80	20
Cefazolin					4	11	29	8	**8**							86.7	13.3
Cefotaxime					56			**4**								93.3	6.7
Colistin			1	10	37	8	1	**2**	**1**							95	5
Enrofloxacin	47	8	2	1	2											100	
Gentamicin				9	29	20	2									100	
Sulfamethoxazole/trimethoprim		53		2	1					**4**						93.3	6.7
Sulfisoxazole													49		**11**	81.7	18.3
Tetracycline					7	9	31	2		**11**						83.3	18.3

S = susceptible, R = resistant. Gray shaded areas indicate the antimicrobial concentration tested, while bold indicates the number of resistant strains according to the CLSI M-100 cut-off values [20].

**Table 2:** Genetic and phenotypic profiles of 24 *E. coli* isolates resistant to tested antibiotics.

ARGs Detected	Phenotypic Resistance	Number of Isolates
*sul1*, *int*1	FIS	4
*tet*(A), *sul1*, *int*1	AMP-TET-SXT-FIS	3
*tet*(A), *tet*(B)	AMP-TET	3
*bla*_TEM_, *bla*_CTXM_, *tet*(A)	AMP-AUG2-FAZ-FOT-COL-TET	2
*bla*_TEM,_ tet(A), tet(B)	AMP- FAZ-TET	1
*bla*_TEM_, *tet*(A), *tet*(B)	AMP-AUG2-TET-FIS	1
*bla*_TEM_	AMP-AUG2-FAZ	1
*bla*_CTXM_	FAZ-FOT-FIS	1
*sul1*, *int*1	SXT	1
*bla*_CTXM_	FOT	1
*tet*(B)	TET-FIS	1
*	FAZ-COL-FIS	1
*	AUG2-COL	1
*	AMP	1
*	FAZ	2

FIS: sulfisoxazole; AMP: ampicillin; TET: tetracycline; SXT: trimethoprim/sulfamethoxazole; AUG2: amoxicillin/clavulanic acid; FAZ: cefazolin; FOT: cefotaxime; COL: colistin; * No ARGs detected.

## Data Availability

All data discussed are contained in the manuscript.

## Supplementary Materials

The following supporting information can be downloaded at: https://www.mdpi.com/article/10.3390/vetsci10120680/s1; Table S1: Real-Time PCR primers for ESBL profile determination; Table S2: Target ARGs detected, primer pairs and annealing temperature used.
