# Supplementary material for: Antibiotic Resistance Genes Carried by Commensal Escherichia coli from Shelter Cats in Italy
Source: Vet Sci. 2023 Nov 28;10(12):680. doi: 10.3390/vetsci10120680 (PMC10747167; doi:10.3390/vetsci10120680)
Supplement: Supplementary file 1 [file vetsci-10-00680-s001.zip › vetsci-2695487-SI.pdf]

Supplementary Materials Table S1. Real-Time PCR primers for ESBL profile determination

Target gene		Primer sequence (5′→3')
	Forward	GCATCTTACGGATGGCATGA
*bla*TEM	Reverse	GTCCTCCGATCGTTGTCAGAA
	Probe	FAM-CAGTGCTGCCATAACCATGAGTGA-BHQ-1
	Forward	TCCCATGATGAGCACCTTTAAA
*bla*SHV	Reverse	TCCTGCTGGCGATAGTGGAT
	Probe	Cy5-TGCCGGTGACGAACAGCTGGAG-BBQ-650
	Forward	CGGGCRATGGCGCARAC
*bla*CTXM_A	Reverse	TGCRCCGGTSGTATTGCC
	Probe	FAM-CCARCGGGCGCAGYTGGTGAC-BHQ1
	Forward	ACCGAGCCSACGCTCAA
*bla*CTXM_B	Reverse	CCGCTGCCGGTTTTATC
	Probe	Yakima Yellow- CCCGCGYGATACCACCACGC-BHQ1
	Forward	GGCAAACAGTGGCAGGGTAT
*bla*CMY	Reverse	AATGCGGCTTTATCCCTAACG
	Probe	ROX-CCTACCGCTGCAGATCCCCGATG-BHQ-2

Supplementary Materials Table S2. Target ARGs detected, primer pairs and annealing temperature used

Antibiotic	Target gene	Primer sequence (5′→3')	Amplicon size (bp)	Annealing temperature (°C)	References
Tetracyclines	*tet(A)*	GCTACATCCTGCTTGCCTTC<br>CATAGATCGCCGTGAAGAGG	210	60	[19]
	tet(B)	TTGGTTAGGGGCAAGTTTTG<br>GTAATGGGCCAATAACACCG	659	60	
Sulphonamides	*sul1*	TCACCGAGGACTCCTTCTTC<br>AATATCGGGATAGAGCGCAG	316	50	[20]
	sul2	TCCGGTGGAGGCCGGTATCTGG<br>CGGGAATGCCATCTGCCTTGAG	191	58	[21]
Fluoroquinolones	*gyrA*	AAATCTGCCCGTGTCGTTGGT<br>GCCATACCTACGGCGATACC	344	60	[22]
	parC	CTGAATGCCAGCGCCAAATT<br>GCGAACGATTTCGGATCGTC	188	60	
Class 1 integron	*int1*	GGCTTCGTGATGCCTGCTT<br>CATTCCTGGCCGTGGTTCT	148	55	[19]
